# Role of intercellular interactions on single cell and population level responses: considerations for multicellular bioreporter design

**DOI:** 10.3389/fmolb.2025.1595363

**Published:** 2025-10-13

**Authors:** Douglas Lin, Michael Martin

**Affiliations:** ^1^ Math Science Technology Magnet, duPont Manual High School, Louisville, KY, United States; ^2^ Micro/Nano Technology Center, University of Louisville, Louisville, KY, United States

**Keywords:** Gillespie algorithm, stochastic simulation, bioreporter, validation, multicellular, unicellular

## Abstract

**Introduction:**

Bioreporters are genetically engineered cells that produce detectable responses in the presence of specific analytes, providing a cheap, mass-producible, and accurate method of analyte detection. Most research focuses on the single cell-level, where all engineering is concentrated on the interactions within a single cell. However, intercellular communication is a well-known natural phenomenon that has been associated with sensitive responses to certain chemical stimuli, yet incorporation of intercellular communication into bioreporter design is exceedingly rare and the effect of intercellular signaling on single-cellular and population level responses has not been explicitly characterized before.

**Methods:**

In this work, a multicellular simulator implementing the Gillespie algorithm and compatible with Virtual Cell-designed networks is created and used to demonstrate nuances to multicellular stochastic simulations. The algorithm was used to simulate multiple cells in a reaction network in which a self-promoting and membrane permeable transcription factor also induces production of a cell-bound reporter protein. A proof-of-concept bioreporter that responded to an environmental analyte while leveraging intercellular interactions for signal production was also designed and simulated for 50 s of simulation time. Simulated systems with multiple cells were compared to single-cellular simulations.

**Results:**

Simulations for 20 s of simulated time show that while final reporter protein count per cell decreased as cell count increased, aggregate final reporter protein number across all cells significantly increased. Interestingly, 50 s simulations show final reporter protein count per cell significantly increasing as the number of cells increases. Greater number of bioreporter cells resulted in significantly greater amounts of signal protein produced in response to the same amount of starting analyte both on the population level and on the individual cellular level.

**Discussion:**

The results show significant differences between the results of multicellular and single cellular simulations, which demonstrates the importance of simulating multiple cells to obtain nuanced results. The amplification of signal protein produced by an increasing number of simulated bioreporter cells indicates great potential for multicellular bioreporter designs to amplify response magnitude and sensitivity on the individual cellular level. These results warrant further research into the application of different simulation algorithms and multicellular bioreporter design and modeling.

## Graphical Abstract

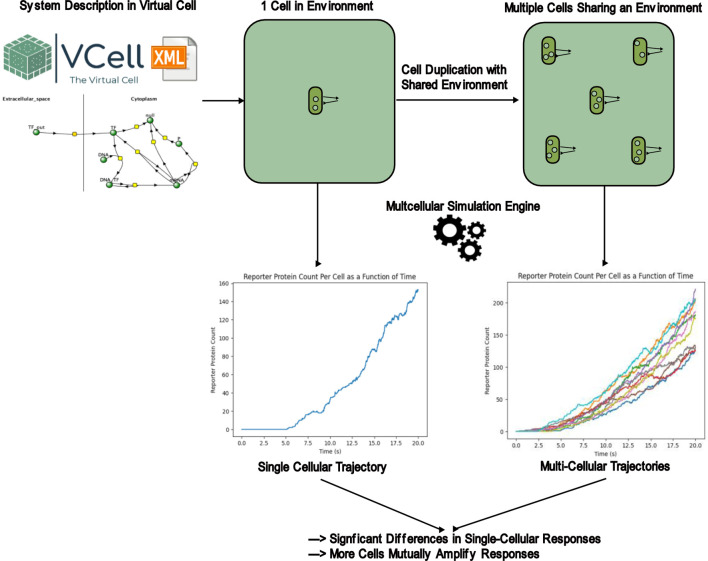

## Introduction

Cellular simulation is mainly concerned with accurately modeling the rates at which intracellular processes, such as transcription and translation, occur. Accurate simulation of these reactions over time allows for accurate understanding of cell behavior without costly lab expenses, saving long term costs. These reactions that occur on subcellular scales cannot be accurately modeled by differential equations due to extremely low concentrations of reactants. Algorithms that incorporate the stochastic effects dominant at such low concentrations are required. Indeed, such stochastic effects explain behavior of transcriptional networks not captured by deterministic models (Hahl and Kremling, 2016) and help elucidate biological design features to manage stochastic noise that would other not be appreciated (Boettiger, 2013). In a comparative study by Johnson et al., in 2021, they found that in biochemical networks with feedback loops produced major differences in results when simulated with stochastic dynamics when compared to deterministic simulations (Johnson et al., 2021).

A common method of simulating these processes is using the Gillespie algorithm (Gillespie, 1977). In a system with many possible reactions, the Gillespie algorithm first computes the instantaneous reaction rates of every reaction. The algorithm then chooses a reaction to occur next based on its relative reaction rate. Finally, the time it takes for that reaction to occur is drawn from an exponential distribution with λ equal to the sum of the instantaneous reaction rates of all reactions. The Gillespie algorithm is the most fundamental and exact method to predict the future evolution of a biochemical system while considering stochastic effects (Warne et al., 2019). The Gillespie algorithm, or an algorithm mathematically equivalent to it, is very commonly used in many stochastic simulation tools, most of which are designed for single-cell simulations. Examples include Virtual Cell (Resasco et al., 2011) and COPASI (Hoops et al., 2006). While these simulators can accurately describe single cell behavior, they are not equipped to simulate multicellular systems at scale. Their inability to model multicellular systems prevent possible effects of intercellular interactions from being understood. However, the effect of intercellular interactions in stochastic simulations has not been explicitly characterized before, so the knowledge lost from the inability to model multicellular systems is not well understood either. This project aims to fill this gap in knowledge by explicitly comparing the results of a single cellular simulation with the results of a multicellular simulation, both on the single cellular and population level response. The research question for this project is: do intercellular interactions significantly affect single cellular and population level responses? The hypothesis for this project is that if intercellular interactions are accounted for, then the results produced by multicellular simulations with intercellular interactions will be significantly different from the results produced by a unicellular simulation that does not account for intercellular signaling, both on the individual cellular level and on the population level. To achieve this, the results of multicellular simulations with intercellular interactions will be compared to the results of an unicellular simulation. The exact method of quantifying and comparing results of different simulations is described in the Methodology section.

A better understanding of the role of intercellular signaling will be valuable in multiple areas within biology. For example, construction of genetically engineered whole-cell biosensors, which are meant to produce detectable signals in response to the presence of certain environmental analytes, is a current area of active research (Saltepe et al., 2017). These biosensors are constructed with consideration only within a single cell and the processes within it. These biosensors can often be modeled in single-cellular cell simulators like Virtual Cell and validated for responses to their intended stimuli before being expressed in a lab, saving cost. However, a better understanding of intercellular interactions in cellular responses could allow for the construction and computational validation of biosensors that leverage intercellular signaling, expanding the possible areas for biosensor optimization. Intercellular signaling among bacteria has long been known and studied in nature, especially in the context of propagating responses to a stimulus to an entire colony of bacteria (Antunes and Ferreira, 2009). Adapting this natural process would likely yield better biosensor designs than designs that only focus on intracellular interactions due to the proven effectiveness of intercellular interactions in nature. Coordinated luminescence is already naturally achieved through quorum sensing (Li and Nair, 2012). Biosensors are cheap, onsite detection methods for soil and water contaminants (Zeng et al., 2021), so the construction of more sensitive biosensor designs could further improve the usefulness of this detection method. Cheaper detection methods could allow for higher throughput and more frequent testing for dangerous contaminants, potentially saving lives by finding sources of contamination early before the effects of these contaminants manifest severely. By stochastic simulations to model intercellular communication, a secondary research hypothesis explored in this project is as follows: a bioreporter design incorporating intercellular signaling will produce a greater signal in response to a selected analyte.

One of the most popular cellular simulation tools is Virtual Cell, created by Resasco et al., in 2011. Virtual Cell has a graphical user interface for designing reaction networks and uses a modified but mathematically equivalent version of the Gillespie algorithm to model the evolution of all species present in the user-created reaction network according to the parameters set by the user. It also allows users to define different compartments with different volumes to separate interactions within a cell and those outside. The user-friendly interface and ease of use makes Virtual Cell very useful for simulating reactions occurring in a small number of compartments, such as the reactions in a single cell and an extracellular space. However, Virtual Cell does not have any built-in capability to extend simulations to multiple cells easily. It is possible to manually create multiple compartments with duplicated reaction rules to represent multiple cells, however the process is tedious and not feasible for simulating hundreds of cells, despite many real-life situations containing hundreds or thousands of cells. This project aims to extend simulation capabilities using the Gillespie algorithm to a large number of cells while also accounting for shared chemical species between cells to represent intercellular interactions, then comparing the changes in simulation results that result from multicellular simulations that would otherwise not occur in the single cellular simulations that Virtual Cell is most used for.

Previous research on simulating intercellular interactions have taken diverse approaches. Weber and Buceta in 2013 simulated a biological “switch” comprising 1000 cells and accounting for the production and effects of intercellular quorum sensing molecules produced by each of the cells. Each cell contains the genetic components for a positive feedback loop where extracellular quorum sensing molecules diffuse into cells and stimulate the production of a reporter protein and more quorum sensing molecules. These interactions are very similar to the ones that will be used in this project, as described in the Methodology section. Weber and Buceta individually plotted the concentrations of select chemical species in each cell simulated and observed that significant variations in reporter protein concentration between cells existed. Given enough time all cells plateaued in reporter protein production. They attributed their observations to the impact of intercellular signaling, however they did not explicitly compare the simulation results of the population of cells with a simulation of only a single cell. They also did not vary the amount of cells they simulated to observe the effect of the number of cells in a population on the behavior of the individual cells or the population of cells as a whole. Therefore, the actual impact of intercellular interactions on their simulation results is not clearly defined. This project seeks to explicitly compare the difference in results caused by accounting for intercellular signaling using a similar reaction network as the one used by Weber and Buceta to determine if the presence of multiple cells and their intercellular interactions contributed significantly to the behavior they observed. This project also creates a general framework to simulate any multicellular system based off of one designed in the Virtual Cell graphic user interface. Finally, this project extends beyond the work of Weber and Buceta by analyzing generalized constructions of multicellular signaling and bioreporters for analytes.

Duso and Zechner in 2020 studied multicellular simulations from the perspective of a multi-compartmental system. Intercellular interactions were represented as cells directly acting on each other. The authors developed differential equations to accurately capture summary statistics of trajectories generated by stochastic simulation. While their work focuses solely on the time evolution of multicellular systems, this work compares multicellular systems with single cellular ones. Additionally, this work simulates the mechanism of intercellular communication, i.e., the diffusion of a signaling protein, instead of abstracting interactions as probabilistic events.

Instead of explicit simulation of multiple cells, Zhou et al., in 2005 modeled intracellular and extracellular noise of a synthetic quorum sensing circuit from the perspective of a single cell using Langevin equations. In this work, we explicitly model multiple cells sharing the same environment to determine similarities and differences between their findings and ours. Additionally, we vary the cell population size to examine its effect on cellular responses.

Smith and Grima in 2018 use stochastic simulation to compare independent, insulated cells with tissue-bound cells able to communicate with each other. Their independent cells are analogous to a single cell simulation, and their tissue-bound cells are analogous to a multicellular system. However, they do not include a shared extracellular space, and this work includes an additional simulation of a bioreporter design.

This work focuses on the explicit comparison of a given biochemical reaction system between a single cellular system and a multicellular system to establish a significant difference. Then, the findings are extended to a proof-of-concept model of a bioreporter design that leverages multicellular interactions to increase signal strength. In the process, a general framework to simulate multicellular systems based off of Virtual Cell-designed single-cellular systems is created.

## Methodology

### Algorithm overview

**FIGURE 1:**
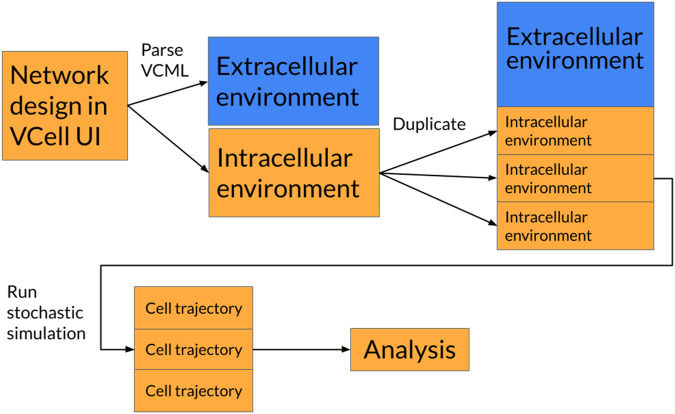
Diagram of how unicellular simulations are converted into multicellular systems. “VCell” is the abbreviation for the Virtual Cell software. VCML is the file extension used by VCell to represent simulation environments.

In order to simulate multiple cells, a custom simulator first had to be created because none currently exist. A diagram of how multicellular simulations are constructed can be seen in Figure 1. The simulator implements the Gillespie Stochastic Simulation Algorithm, which probabilistically simulates individual reactions based on their relative rates as well as the time between reactions (Gillespie, 1977). The rate, or activity, of a given reaction is given by the elementary rate equation

$$a_{j}=k_{j}\;*\;N_{Reactant\;1}\;*\;N_{Reactant\;2}\;*\;\ldots \;N_{Reactant\;n}$$

where *N*_*Reactant n*_ is the number of molecules of *Reactant n* that are present in the system, and *k*_*j*_ is the reaction rate corresponding to reaction *j* for 1 ≤ *j ≤ m*, where *m* is the total number of reactions defined for a system.

Simulation time is incremented by discrete time step τ seconds, which is sampled from the exponential distribution with a probability density function of

$$p\left(\lambda \right)=\lambda e^{-\lambda \;x}\text{ and }\lambda =\sum_{j=1}^{m}a_{j}$$

Before each time increment, a reaction is chosen to occur by the algorithm by finding a number *k* 1 ≤ *k ≤ m* that satisfies the inequality

$$\sum_{j=1}^{k}a_{j}<∼U\left(0,\sum_{j=1}^{m}a_{j}\right)<\sum_{j=1}^{k+1}a_{j}$$

Where *∼ U(a, b)* represents a sample from the Uniform Distribution with a lower bound of *a* and an upper bound of *b*. This essentially selects a reaction at random, with each reaction weighted by its activity. The *k*th reaction is then simulated, and the number of molecules of reactants and products of the reaction are appropriately incremented or decremented. The process is repeated until simulation time exceeds the allotted amount of time.

The algorithm was implemented in the Python (3.11.8) programming language and uses the Numba (v0.59.0) and Numpy (v1.26.0) libraries for runtime optimization. All code was written in a Jupyter notebook and run locally on a Macbook M2. Multicellularity was achieved by duplicating the intracellular cellular environment so that they all shared the same species in the extracellular environment. Thus, we reproduce a homogenous cell population within a shared environment.

All code and supporting files used to generate the results in this work are hosted on GitHub and may be accessed in Supplementary Appendix A.

### Description of simulated cells and reaction networks

In this work we simulate an idealized, theoretical bacterial cell that is defined only by the biochemical reaction pathways of interest. As bioreporter pathways should be designed to be biologically orthogonal to native pathways, this simplification should be representative of real circumstances (Costello and Badran, 2021). Similarly, the environment that the cells are placed in is also defined only with the species of interest that interact with the simulated biochemical pathway. In future works, more chemical species and interactions can easily be defined within cells and the shared environment.

Any reaction that can be characterized with a set of reactants and products can be modeled with this simulator. In this work we model protein-DNA reversible binding, translation and transcription, protein degradation, ligand-protein reactions, and diffusion through membranes. Given the generality of what can be simulated, more types of reactions can be defined and simulated, e.g., small molecule-small molecule interactions, protein-protein interactions, RNA-DNA interactions, and more.

**FIGURE 2:**
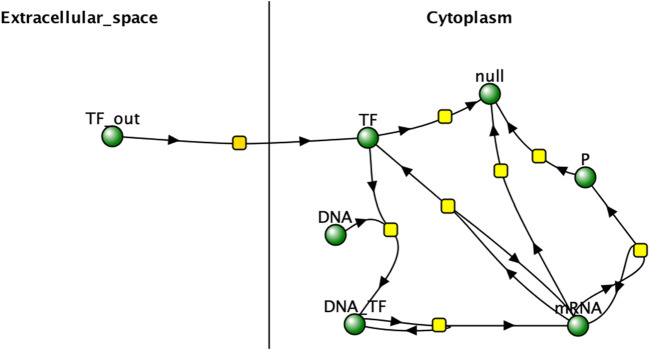
Virtual Cell network representation of experimental reaction network. “TF” represents the reporter protein inside the cell and “TF_out” represents the protein in the extracellular space. Arrows can represent forward and reverse reactions, as is the case with TF_out diffusing into the cytoplasm of each cell and TF diffusing back out into extracellular space. null represents the products of degradation. Image produced by researcher.

The reaction network used in the project was then designed in Virtual Cell (v7.7.0.9) and can be viewed in Figure 2.

In the reaction network, the expression of reporter protein “P” is increased by the concentration of a transcription factor “TF”, which also induces expression of itself. TF binds reversibly to cellular DNA to create a complex that creates mRNA for P and TF that are translated into the proteins. TF is produced within a cell and diffuses freely through the cell membrane, acting as the main mechanism of intercellular interaction. P is created in the cell but cannot diffuse out and is used as the quantification metric to test the hypothesis. This network is very similar to the one described by Weber and Buceta in 2013 and has very clear intercellular interactions, making it a good model for understanding the impact of intercellular interactions on cell responses. It assumes a well-mixed system and no changes in cell population for the duration of the simulation, which is consistent with the real-world use-case of bulk signal production by bioreporter cell populations. It is also consistent with the assumptions in related works (Weber and Buceta, 2013; Duso and Zechner, 2020).

Reaction stoichiometries, rate laws, rate constants, and starting concentrations were then defined. Rate constants were chosen as reasonable estimates rather than modeling an existing biological system. This choice was made to maintain a simple system, and because this work focuses solely on the relative differences between multicellular and unicellular dynamics, thus making the exact parameter values less critical to answering research questions. The rate at which TF diffuses out of the cell was purposefully set to be 100 times faster than the rate at which TF diffuses into the cell. This ratio is the result of a cell that has 1/100 of the volume of its environment. The starting amount of TF_out was set to be 3000 molecules and the amount of DNA was set to be 100 molecules. The starting amount of extracellular TF was estimated to be small enough so that cellular production of TF would be relatively significant compared to the amount of already existing TF, but large enough to have an impact on cellular response. The starting amounts of all other species were set to 0 molecules.

The simulator was then run with these conditions, simulating 1, 10, 25, 50, 75, and 100 cells, with the number of cells acting as the independent variable and the results of the single cellular simulation being the control against which the multicellular simulations will be compared. For each number of cells, two simulations were run for 20 s and 50 s for 50 trials each. 20 s and 50 s were chosen as the simulation time limits primarily to expedite experimentation, as the values provided distinct results while allowing sufficient observation of the evolution of the simulated systems.

The amount of reporter protein P was recorded for every cell after 20 s and 50 s of simulation time. To find the average amount of P present in a cell at 20 and 50 s for a given number of cells simulated, the amount of P in each cell was averaged across all cells and all trials before being recorded. The aggregate amount of P present in all cells at 20 and 50 s for a given number of cells simulated was similarly found by summing the amount of P present in each cell across the number of simulated cells and averaged across trials before being recorded. Both the average amount of reporter protein present in each cell and the total amount of reporter protein present across all cells were the dependent variables used to test the hypothesis.

2-tailed t-tests were performed to determine if multicellular simulations resulted in levels of P protein that were significantly different from the results produced by the unicellular simulation using a significance level of 0.001. A parametric t-test was determined to be appropriate due to the high number of replicates per experimental condition. A threshold of 0.001 was chosen instead of 0.05 to be more stringent on significance given the inherently noisy data being analyzed. These data points will be analyzed in the Data and Results section as well as in the Discussion section. Simulation times have also been compiled and reported in the Data and Results section.

For a proof of concept, a more complex multicellular bioreporter network was designed and simulated with different numbers of cells for 50 s to discern if a system with multiple shared chemical species across cells would behave similarly to a system with only one shared species, as in the experimental reaction network. A shared “signal” molecule is produced by the cells which is induced by the “analyte” and in turn induces the production of a “reporter” molecule. The reporter molecule is used to determine the signal and response strength of the detector cells. The reaction network is displayed as a Virtual Cell network in Figure 2.

## Data and Results

### 20-Second simulations

**FIGURE 3:**
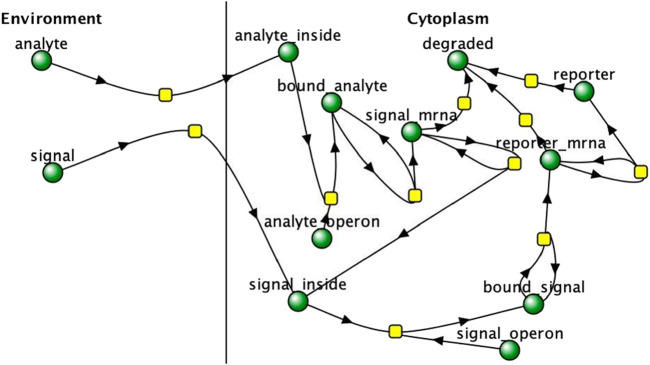
multicellular bioreporter design with two shared chemical species, “signal” and “analyte”. Simulations were run for 50 s of simulation time. Network and reaction rate constants can be found in the public Virtual Cell model called “Bioreporter-POC”. Image produced by researcher.

After 20 s of simulation time, the amount of reporter protein molecules present in each cell was averaged across trials and across all cells simulated. Those values are represented in this table. A 2-tailed t-test was performed on the results of simulating more than 1 cell and the results of a unicellular simulation to establish a statistically significant difference in the results of multicellular simulations compared to unicellular simulations. Only the 10 cell simulation produced results with a p value greater than 0.001, with the 25, 50, 75, and 100 cell simulations producing results with a p value less than 0.001. This means the null hypothesis that multicellular simulations produce insignificantly different results than single cellular simulations can be rejected for all the multicellular simulations that simulate more than 25 cells that were tested. This strongly indicates that multicellular simulations produce statistically significantly different results than single cellular simulations at the single cell level, at least at 20 s of simulation time and for simulations with more than 25 cells. A scatter plot of the data in Table 1 is represented in Figure 3.

**TABLE 1:** Summary statistics of average reporter protein count per cell after 20 s of simulation time.

Cells	1	10	25	50	75	100
Average number of reporter proteins per cell	180.9	174.5	154.7	139.4	124.2	114.0
Standard deviation	45.3	13.0	8.6	6.5	5.1	5.1
2-tailed t-test statistic compared to unicellular result	N/A	1.0	4.2	6.7	9.2	10.9
p-value	N/A	0.3	<0.001	<0.001	<0.001	<0.001
Significant?	N/A	FALSE	TRUE	TRUE	TRUE	TRUE

The data represented in the graph demonstrates a negative, nonlinear relationship between the number of cells simulated and the amount of reporter protein present at the end of simulation time. This trend could be rationalized by considering how additional cells will dilute the intercellular signal amongst more cells, leading to less signal present in each individual cell and therefore eliciting less of a response from each cell.

**FIGURE 4:**
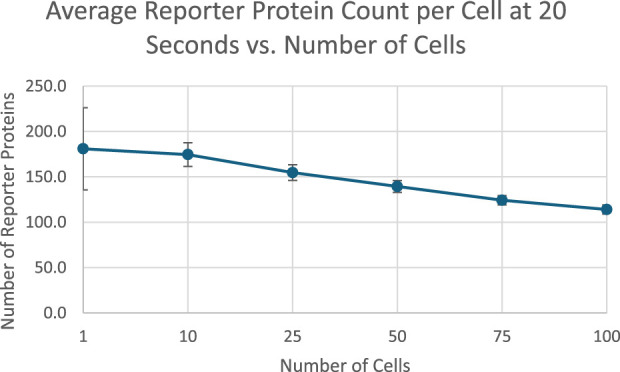
Scatterplot of average reporter protein count per cell at end of simulation time as a function of number of cells simulated, with 1 SD error bars. Image produced by researcher.

At 20 s into simulation time, the amount of reporter protein molecules present in each cell was summed across all cells simulated, then averaged across trials. Those values are represented in this Table 2 tailed t-tests were similarly performed to establish a statistically significant difference in the results of multicellular simulations compared to unicellular simulations. All t-tests resulted in p-values that were multiple orders of magnitude less than the required threshold for significance, p < 0.001, which means the null hypothesis that multicellular simulations produce insignificantly different results than single cellular simulations can be rejected for all multicellular simulations tested. This strongly indicates that multicellular simulations produce statistically significantly different results than single cellular simulations at the population level, at least at 20 s of simulation time. A scatter plot of the data in Table 2 is represented in Figure 4.

**TABLE 2:** Summary statistics of aggregate reporter protein produced across all simulated cells after 20 s of simulation time.

Cells	1	10	25	50	75	100
Aggregate reporter protein count	184.5	1743.7	3855.2	6920.8	9208.1	11208.5
Standard deviation	45.3	129.5	215.7	327.2	380.9	505.6
2-tailed t-test statistic compared to unicellular result	N/A	82.1	120.2	147.1	169.7	156.6
p-value	N/A	<0.001	<0.001	<0.001	<0.001	<0.001
Significant?	N/A	TRUE	TRUE	TRUE	TRUE	TRUE

To show that the pattern shown in Figure 3 was robust to starting TF concentration, we re-ran the simulation outlined in Figure 3 with different starting extracellular concentrations of TF. We found the decreasing pattern held.

The data show a positive, nonlinear relationship between the number of cells simulated and the total number of reporter protein molecules produced across all cells. The positive trend appears to be a consequence of the fact that there are more cells present that can produce the reporter protein, and the nonlinearity could be attributed to the dilution effect that decreases individual cellular response shown in Figure 3. Further analysis can be found in Supplementary Appendix E.

### 50-Second simulations

**FIGURE 5:**
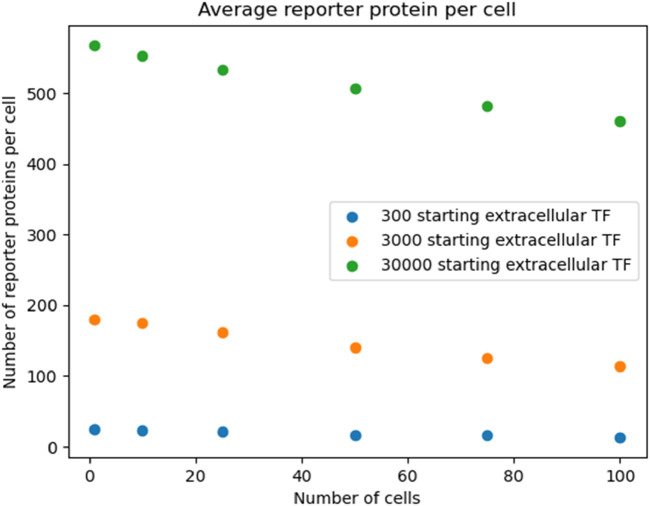
Reproducing Figure 3 with logarithmic scan of the number of starting extracellular TF.

After 50 s of simulation time, the amount of reporter protein molecules present in each cell was averaged across trials and across all cells simulated. Those values are represented in this table. The full plots of the number of reporter protein molecules as a function of time per cell can be seen in Supplementary Figures B1 to B4. A 2-tailed t-test was performed on the results of simulating more than 1 cell and the results of a unicellular simulation to establish a statistically significant difference in the results of multicellular simulations compared to unicellular simulations. All t-tests produced p-values below the required threshold for significance, p < 0.01, which means the null hypothesis that multicellular simulations produce insignificantly different results than single cellular simulations can be rejected for all multicellular simulations tested. This strongly indicates that multicellular simulations produce statistically significantly different results than single cellular simulations at the single cell level, at least at 50 s of simulation time. However, the direction of difference between multicellular and single cellular simulations appears to be reversed for 50 s simulation results compared to 20 s simulation results, with the amount of reporter protein present in each cell increasing as the number of cells simulated increases. A scatter plot of the data in Table 3 is represented in Figure 5.

**TABLE 3:** Summary statistics of average reporter protein count per cell after 50 s of simulation time.

Number of cells	1	10	25	50	75	100
Average number of reporter protein molecules per cell	657.0	791.2	881.7	926.7	937.2	936.0
Standard Deviation	110.9	35.6	30.5	18.5	15.5	15.3
2-tailed t-test statistic compared to unicellular result	N/A	8.14	13.8	17	17	17
p-value	N/A	<0.001	<0.001	<0.001	<0.001	<0.001
Significant?	N/A	TRUE	TRUE	TRUE	TRUE	TRUE

The data show a positive, nonlinear relationship between the number of cells and the number of reporter proteins present in each cell. The trend is initially increasing likely due to the increased cumulative production of the intercellular signal over time by more cells, counteracting the short run effects of dilution. The plateau could be caused by reaching the maximal production rate of the reporter protein, as production rate is limited by the amount of DNA in the cell that can transcribe mRNA to express both the intercellular signal and the reporter protein. The amount of intercellular signal produced by more cells may have completely saturated the available DNA in all cells.

**FIGURE 6:**
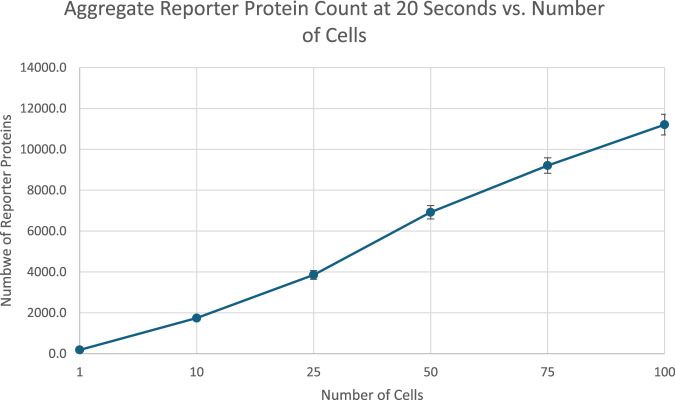
Scatterplot of aggregate number of reporter protein molecules present at 20 s into simulation time as a function of number of cells simulated. Image produced by researcher.

After 50 s of simulation time, the total amount of reporter protein molecules present across all cells was averaged across trials. Those values are represented in this table. A 2-tailed t-test was performed on the results of simulating more than 1 cell and the results of a unicellular simulation to establish a statistically significant difference in the results of multicellular simulations compared to unicellular simulations. All t-tests produced p-values below the required threshold for significance, p < 0.001, which means the null hypothesis that multicellular simulations produce insignificantly different results than single cellular simulations can be rejected for all multicellular simulations tested. This strongly indicates that multicellular simulations produce statistically significantly different results than single cellular simulations at the population level, at least at 50 s of simulation time. A scatter plot of the data in Table 4 is represented in Figure 6.

**TABLE 4:** Aggregate number of reporter protein molecules produced across all cells after 50 s of simulation time.

Number of cells	1	10	25	50	75	100
Aggregate number of reporter protein molecules	657.0	7912.0	22041.6	46332.6	70289.0	93599.5
Standard Deviation	110.9	356.0	762.4	924.6	1159.6	1533.5
2-tailed t-test statistic compared to unicellular result	N/A	137.6	196.3	346.8	422.7	427.4
p-value	N/A	<0.001	<0.001	<0.001	<0.001	<0.001
Significant?	N/A	TRUE	TRUE	TRUE	TRUE	TRUE

Similarly, in the 50 s simulations the pattern of increasing reporter proteins was shown to be robust.

The data show a positive, mostly linear relationship between the number of cells and the total amount of reporter protein produced across all cells. The positive relationship is likely from the increased capacity to produce reporter protein amongst more cells, while the linearity is likely the result of individual cells reaching maximal protein production rate such that the only factor influencing aggregate protein production is the total amount of DNA in the entire population, which scales linearly with the number of cells simulated. Further analysis can be found in Supplementary Appendix E.

### Proof-of-concept bioreporter simulation

**FIGURE 7:**
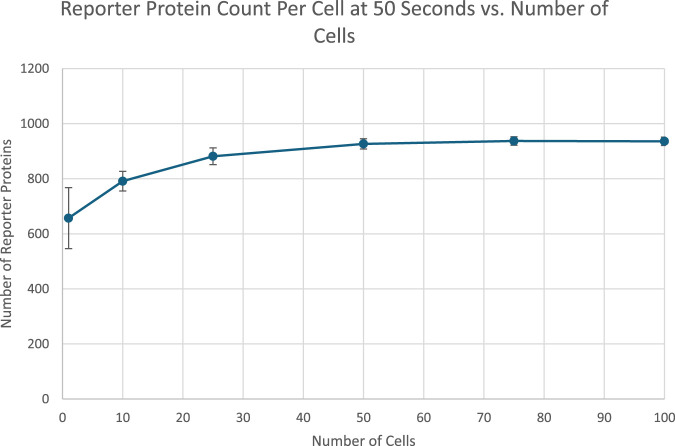
Scatterplot of the average amount of reporter proteins present in each cell at the end of simulation time. Image produced by researcher.

The results of a proof of concept bioreporter designed to leverage intercellular communication is summarized in Table 5 and Figures 7, 9. The design of the bioreporter is detailed in Figure 3. As shown, the amount of reporter protein increases as the number of detector bioreporter cells increases despite the starting analyte count remaining constant across experiments, demonstrating how a quorum of cells increases the signal strength of each individual cell and of the population as well, even in a more complex system with two shared chemical species across cells.

**TABLE 5:** Results of multicellular bioreporter proof of concept. Simulations were run for 50 s with a starting analyte count of 10000 analyte molecules located in the extracellular environment for three replicates each.

Number of cells	1 cell	5 cells	10 cells
Average number of reporter molecules per bioreporter cell	34406	53841.93333	61537.06667
Standard deviation	2398.107379	477.6396271	180.5846154
Significance compared to 1 cell		p < 0.001	p < 0.001
Aggregate number of reporter molecules across all bioreporter cells	34406	269209.6667	615370.6667
Standard deviation	2398.107379	2388.198135	1805.846154
Significance compared to 1 cell		p < 0.001	p < 0.001

**FIGURE 8:**
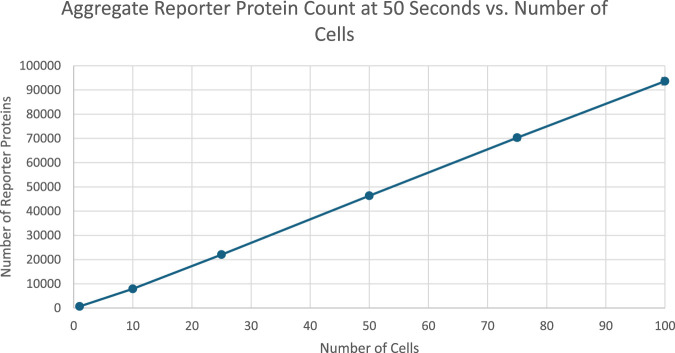
Scatterplot of the aggregate amount of reporter proteins present in each cell at the end of simulation time. Image produced by researcher.

**FIGURE 9:**
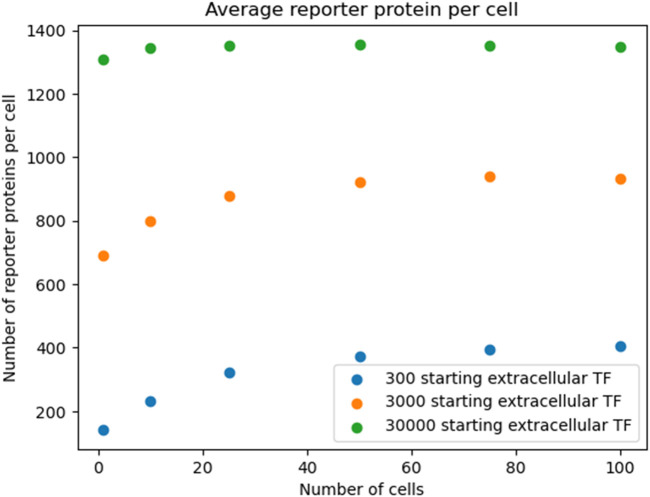
Reproducing Figure 5 with logarithmic scan of starting extracellular TF.

### Runtime analysis

The time required by each simulation has been compiled and presented in this section.

The runtimes for the simulations in the work scale approximately linearly with the number of cells simulated.

## Discussion

The purpose of this project was to investigate the effect of intercellular interactions on the single-cellular and population level responses in kinetic simulations. Multicellular simulations, typically in the context of quorum sensing or morphogenesis, have been explored by various researchers. However, no one has explicitly compared the difference that considering intercellular interactions has on cell- and population-level outcomes compared to only simulating a single cell. There are numerous popular applications for stochastic simulation of unicellular chemical reactions--such as Virtual Cell (Resasco et al., 2011) and COPASI (Hoops et al., 2006)--and it is important to understand how much unicellular simulation results will differ from multicellular simulations. Importantly, cellular simulations can be used for the computational validation of bioreporters, which are genetically engineered cells that produce a detectable signal in response to an environmental contaminant (Zeng et al., 2021). These bioreporters offer a low-cost method for onsite soil and water testing, however most research has focused on the single cell construction of bioreporters. Understanding the role of intercellular interactions on modulating cellular behavior as well as being able to simulate those effects opens a new dimension for the development of multicellular bioreporters to potentially achieve more sensitive detectors, enabling earlier detection of hazardous contaminants.

The hypothesis was that intercellular interactions would produce significantly different results than if they were not accounted for, both on the single cell level and on the population level. To test this hypothesis, a stochastic simulator was implemented to use the Gillespie algorithm to simulate nanoscale chemical kinetics with quantized probabilistic reactions, which more accurately describe chemical kinetics on the scale of single cells than ordinary differential equations (Gillespie, 1977). The simulator was used to simulate a hypothetical reaction network where the expression of reporter protein “P” is increased by the concentration of a transcription factor “TF”, which is also positively regulated by itself. TF is produced within a cell and diffuses freely through the cell membrane, acting as the main mechanism of intercellular interaction. P is created in the cell but cannot diffuse out, making it a useful indicator of transcriptional modulation. Levels of the reporter protein were used as indicators of the effect of intercellular interactions. Using this network, populations of 1, 10, 25, 50, 75, and 100 cells were simulated for 20 and 50 s and the amount of reporter protein present in each cell and in the entire population was recorded at the end of simulation time. The results of those simulations are discussed below.

**FIGURE 10:**
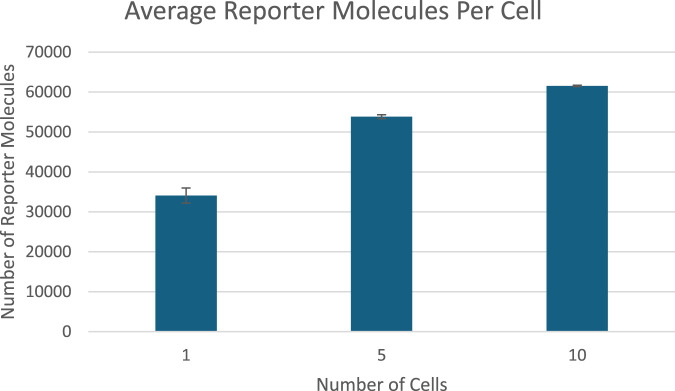
Reporter protein production per cell at the end of 50 s of simulation time graphed against number of detector cells. Image produced by researcher.

In Figure 10, the amount of reporter protein produced in each cell decreases as more cells are simulated, with nearly all multicellular simulations producing statistically significantly different results from the unicellular simulation. This validates the hypothesis that multicellular simulations would produce statistically significantly different results from unicellular simulations at the single cellular level. The reason why reporter protein P production per cell would decrease could be explained by the faster rate of extracellular transcription factor TF depletion. The rate at which TF diffuses into a cell and thereby becoming unavailable to other cells increases as the number of cells increases, as they all share the same extracellular space. Therefore, there would be less intracellular concentrations of TF on average and less upregulation of P expression per cell due to lower TF concentrations. However, it may be possible that in the long run, more TF would be collectively produced by the increased number of cells such that it counteracts the increased rate of extracellular TF depletion.

Figure 2 indicates that the total amount of reporter proteins present in all cells at the end of 20 s of simulation time increases as the number of cells being simulated increases, with each multicellular simulation yielding statistically significant results compared to the unicellular simulation. This validates the hypothesis that multicellular simulations would yield significantly different results than unicellular simulations at the population. This trend is a natural consequence of accounting for more reporter protein to the total amount counted by including more cells in the total amount counted, and the trend is similar to how real bacteria must coordinate with each other to produce a visible glow in certain bioluminescent organisms, as single bacteria produce much less light than a colony (Smith and Grima, 2018). This graph does demonstrate that the dilution effect amongst multiple cells does not outweigh the increased reporter protein production caused by the presence of more cells and therefore greater capacity for reporter protein production. The upward trend suggests multicellular bioreporters are a promising candidate for better bioreporter signal strength. However, the graph does not increase linearly with the number of cells added, as might be naively expected of increasing the capacity for reporter production. The nonlinearity shown in the graph demonstrates the importance of actually simulating multiple cells when considering multicellular systems instead of simulating a single cell and multiplying its results by the number of cells in the system that is supposed to be simulated. If that were done here, the graph of those results would be perfectly linear, and an overestimate of the real results obtained from realistically simulating multiple cells. It is worth noting, however, that simulation of multiple cells is much more computationally intensive than approximations like scaling up the results of a single celled simulation. Future work would include optimizations to the algorithm used in this project for less prohibitive runtimes and better scalability on parallel architectures. Future work would also include comparison of different methods of approximating multicellular simulations with less complexity. For example, an analysis of how the results and runtime of a scaled single cellular simulation differs from a true multicellular simulation under different conditions could be performed. Similar analysis could also be performed on a simulation approximating a multicellular system as one large cell with modified amounts of DNA and membrane diffusion rates. Future work could even include tuning these approximations to find the best balance between accuracy of results and brevity of runtime.

The amounts of reporter protein present in each cell and across all cells were also recorded and analyzed after 50 s of simulation time. In Figure 3, the amount of reporter protein in each cell after 50 s of simulation time is plotted against the number of cells simulated. 2-Tailed t-tests show that every multicellular simulation produced results that were statistically significantly different from the results produced by the single cellular simulation, again validating the hypothesis. Interestingly, the amount of reporter protein present in each cell actually increases as the number of cells simulated increases, a reversal of the trend after 20 s of simulation time. This could be explained by the long term manifestation of the increased capacity to produce the intercellular signal due to the presence of more cells. In fact, the maximal rate of reporter protein production and intercellular signal production appears to have been reached, as indicated by the leveling off of the amount of reporter protein present in each cell for multicellular simulations of more than 25 cells. Greater long term production of the signal TF leads to greater concentrations in the extracellular space, which in turn leads to greater amounts of TF in each cell that elicit a greater response, as is shown in the graph. The effect of multiple cells positively influencing the behavior of single cells in the same population is reminiscent of the results found by Weber and Buceta in their work in simulating a similar positive feedback loop in a colony of cells in which a membrane-crossing molecule produced by each cell induces its own production (2013). Although they did not vary the amount of cells they simulated, they observed that their population of simulated cells, under the influence of an intercellular signal that they each produced, all cells increased their production of that signal up to a maximal production rate. Like in their work, a maximal rate of production was also reached in this project.

Figure 5 represents the total amount of reporter protein present across all cells after 50 s of simulation time. 2-Tailed t tests again showed every multicellular simulation produced statistically significant differences from the single cellular simulation, validating the hypothesis at the population level. The graph increases monotonically as the number of cells simulated increases. When more than 25 cells are simulated, the graph appears to increase a closely linear trend, likely a result of reaching the maximal protein production rate. As a result, the actual trend shape of how much protein is produced across all cells as a function of the number of cells in a population cannot be determined from these results. Future work that aims to further analyze the dynamics of aggregate protein production in a multicellular population need to consider methods to prevent complete saturation of protein production rate. The overall positive trend of the graph is not only similar to the previously discussed results by Weber and Buceta in 2013, but also the real life phenomenon of bacterial colonies using quorum sensing signals to coordinate luminescence (Li and Nair, 2012). Bacteria in colonies coordinate amongst the individual cells to fluoresce more brightly to take advantage of greater colony sizes, producing pulses of visible color.

Finally, the proof-of-concept multicellular bioreporter simulation did exhibit significant signal amplification on the population level and on the single cellular level with two shared chemical species: an analyte, and a cell produced signal protein that also acted as a communication medium. These results validate the secondary research hypothesis that a bioreporter using intercellular interactions would create a stronger signal in response to a given analyte. The proof of concept’s success in amplifying the response of each cell shows great promise for future research in multicellular bioreporter systems.

**FIGURE 11:**
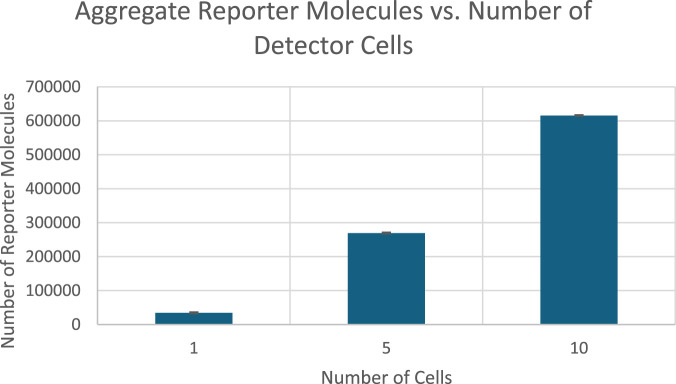
Total Reporter protein production across all bioreporter cells at the end of 50 s of simulation time graphed against number of detector cells. Image produced by researcher.

Figure 11 through 13 show how long each simulation took to complete. As diverse results were obtained with the current experimental parameters, no simulations with more cells or for longer periods of time were executed. However, future work could focus on expanding simulation times and/or cell population sizes. These larger systems would likely necessitate multiple days’ worth of compute-time without significant improvements to algorithmic efficiency or hardware available.

**FIGURE 12:**
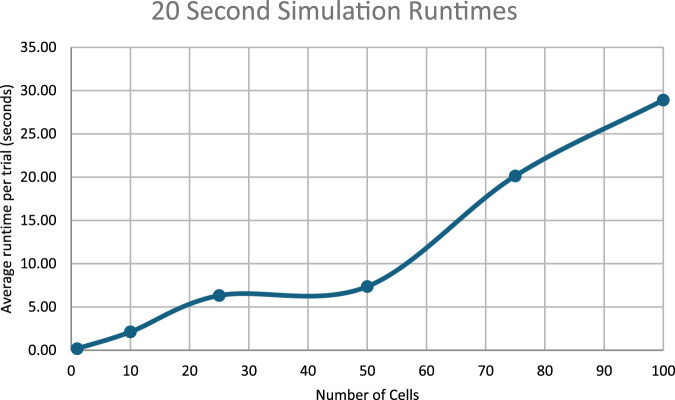
Runtimes of the 20 s simulations.

**FIGURE 13:**
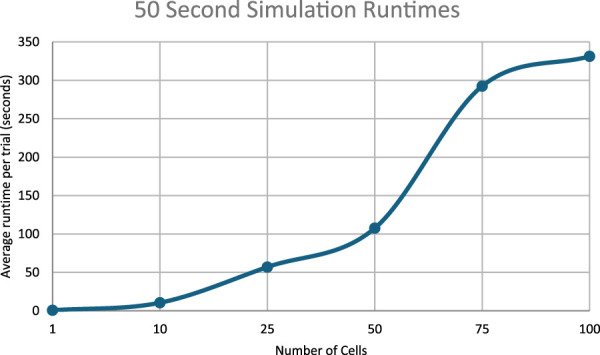
Runtimes of the 50 s simulations.

**FIGURE 14:**
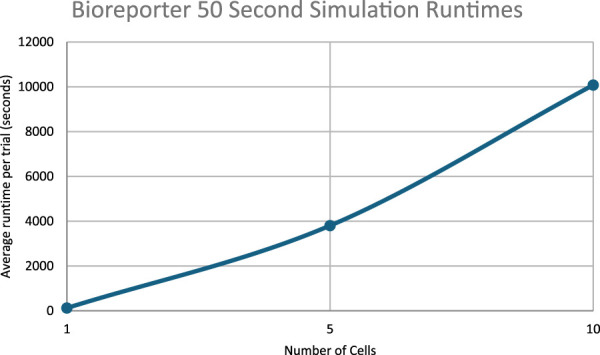
Runtimes of the 50 s bioreporter simulations.

Intriguingly, all simulations resulted in a decreasing standard deviation in average reporter protein concentration per cell as the number of cells simulated increased, even despite increasing numbers of reporter proteins in each cell. This agrees with Smith and Grima’s simulation of a two-stage gene simulation network in independent cells compared to tissue-bound cells (Smith and Grima, 2018). However, the trend appears to contradict Smith and Grima’s simulation of a system in which expression products dimerize. This indicates the networks constructed in this work are more like the former system than the latter. Using the authors’ intuitive explanation, it can be inferred that the constructed networks exhibit substantially more intracellular variability than variability induced through intercellular fluctuation. Similarly, our results also agree with Zhou et al., who found extracellular noise in a quorum sensing circuit served to synchronize cell responses (Zhou et al., 2005). This intracellular response variability in environments of varying cell populations can be qualitatively observed in Supplementary Appendix B.

Overall, simulation data established a significant difference in the results of multicellular and unicellular stochastic simulations in systems where the number of shared species--such as TF in this experiment--is affected by the cell population, thereby strongly supporting the hypothesis established at the beginning of experimentation at both the single cellular and population level. Future work would include improving multicellular simulation software to determine the long-term effects of multicellular simulation compared to unicellular simulation. Future work would mainly be focused on the simulation, design, and computational validation of multicellular bioreporter designs. The preliminary results obtained from this project suggest that leveraging intercellular interactions in multicellular designs is a very promising direction for developing biosensors to emit stronger and potentially more sensitive signals in response to low concentrations of contaminants. The ability to model these interactions allow for the computational validation of novel multicellular bioreporter designs before wet lab work, saving lab expenses and time spent on faulty designs. Thus, future work involves implementing real protein cascade networks with experimentally or computationally determined rate constants for all associated reactions before simulating those networks to design novel multicellular bioreporter designs. Furthermore, given the demonstrated nuances of multicellular simulations, spatial stochastic simulations would likely uncover even more complex patterns in multicellular simulations, where the transient locations of chemical species and cells can influence the progression of a system of chemical reactions (Marquez-Lago and Burrage, 2007). The Smoldyn simulator (Andrews, 2016) is an existing program used by Virtual Cell to implement spatial stochastic modeling, and could be adapted for multicellular simulations much like how Virtual Cell capabilities were adapted for the stochastic simulations described in this work. Another direction of future work would include methods to tackle the “inverse problem” as described by Warne et al. where reaction parameters are obtained through experimental data (Warne et al., 2019). As demonstrated, multicellular systems produce distinct results on the cellular level, so solving the inverse problem will be more complex in systems where multiple cells are interacting. A summary table of challenges of this approach and avenues of future work is listed in Table 6.

**TABLE 6:** Summary of limitations and avenues of future improvement.

Challenge	Avenue of future improvement
Remaining directions for runtime optimization	Implement Gibson-Bruck algorithm, reducing time complexity of each step; parallel simulation of replicate trials
Only supports homogenous cell populations	Implementation of more complex rules for joining compartments in a single simulation for heterogenous cell populations
Simulated in purely theoretical cell environments to demonstrate patterns	Use of empirical rate laws to more accurately model real systems
Static cell population	Implementation of cell population changes during simulations
Assumption of well-mixed system	Implementation of spatiotemporal stochastic modeling

## Data Availability

The raw data supporting the conclusions of this article will be made available by the authors, without undue reservation.
