# Supplementary material for: Role of intercellular interactions on single cell and population level responses: considerations for multicellular bioreporter design
Source: Front Mol Biosci. 2025 Oct 13;12:1595363. doi: 10.3389/fmolb.2025.1595363 (PMC12554554; doi:10.3389/fmolb.2025.1595363)
Supplement: Supplementary file 2 [file DataSheet1.docx]

## Appendix A

Computer Code Used in the Project


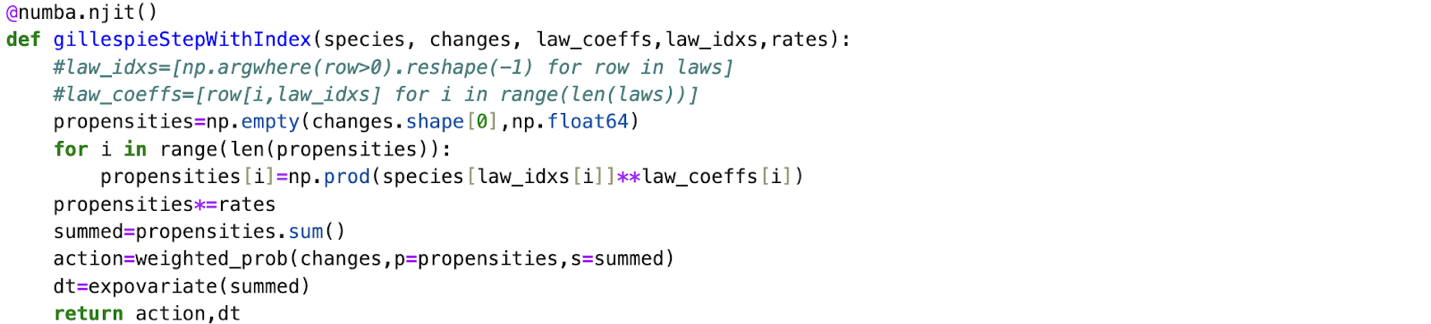


Figure A1: Implementation of the Gillespie algorithm

The Gillespie algorithm was implemented in the programming language python using the library NumPy for optimized mathematical operations and Numba for additional code optimization through Just-In-Time compilation to machine executable instructions.

Figure A2: implementation of multicellular simulation. The Numba library was used to accelerate math operations and parallelize loops.


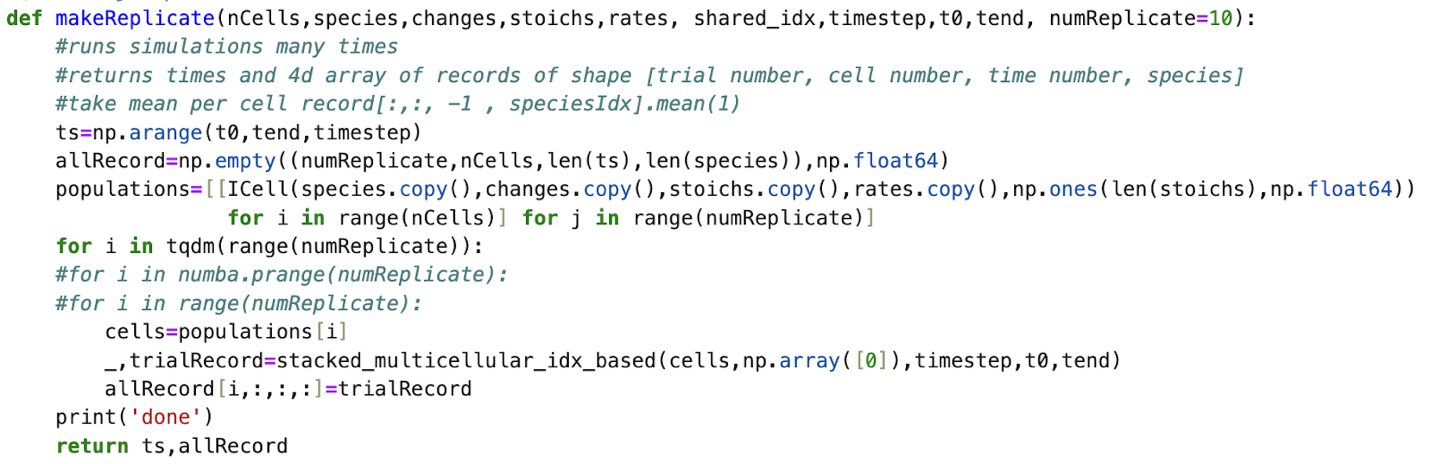


Figure A3: implementation of helper function to easily generate data for multiple trials


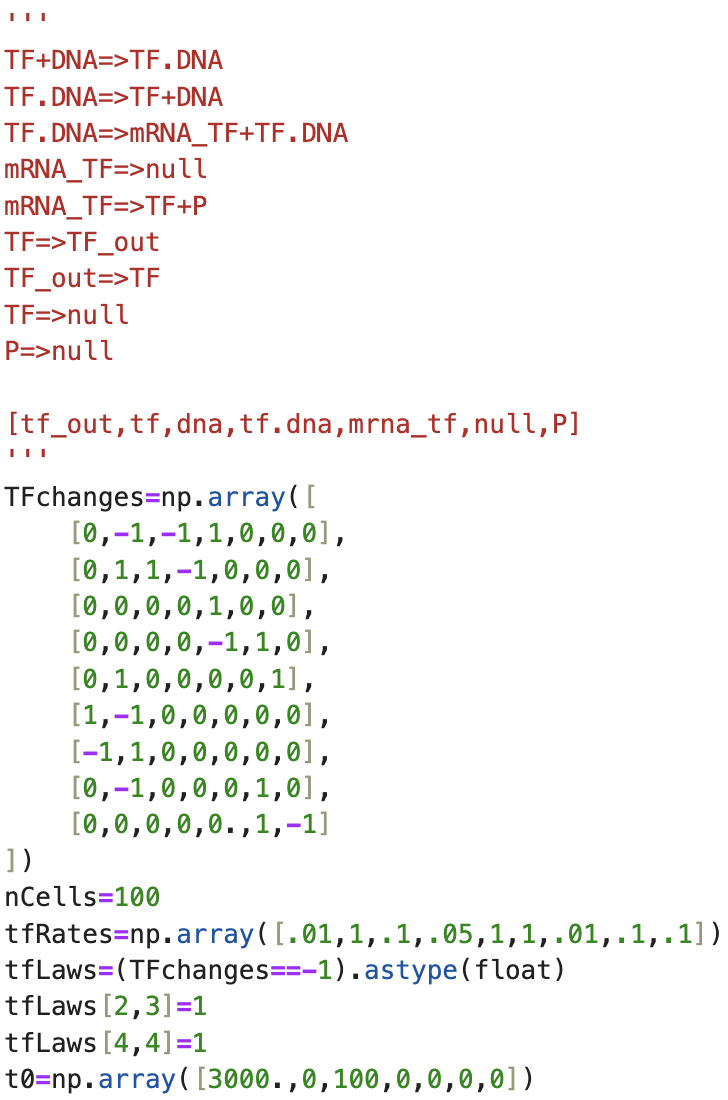


Figure A4: Starting conditions and reaction definitions for each cell

The starting number of molecules of transcription factor TF in the extracellular space is 3000 and each cell starts with 100 molecules of DNA. All other chemical species start at 0. “TFchanges” represents the 9 reaction stoichiometries in the system, with each element describing how a certain chemical species will change when that reaction occurs. “tfRates” is the 9 element array with rate constants for each reaction in “TFchanges”. “tfLaws” is an array that determines how many times a reactant appears in the activity equation for a given reaction. “t0” is the array containing the starting values for each chemical species.


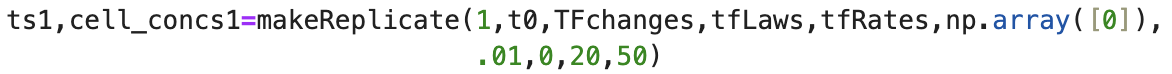


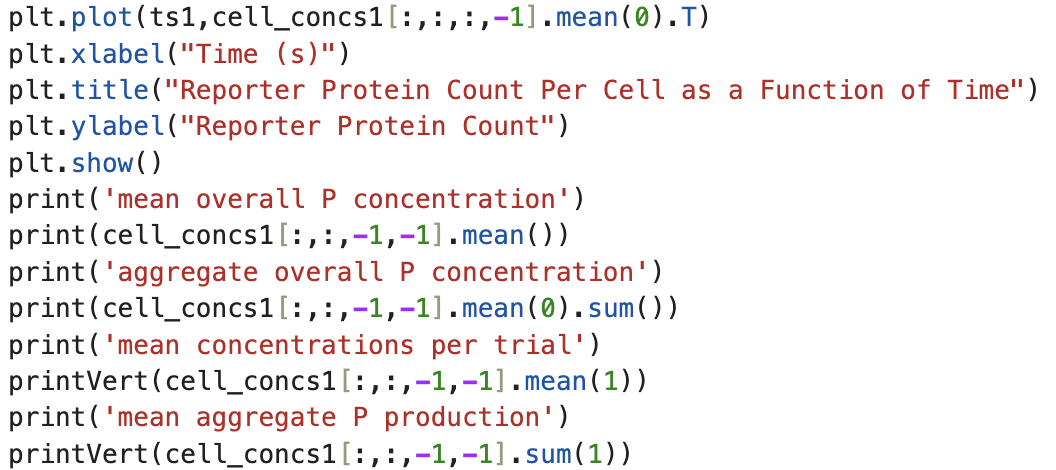


Figure A5: Generation, plotting, and summarization of simulation data found in this paper. Similar code is used for multicellular simulation results but are omitted in the appendices for brevity.

All code used in this work is hosted on GitHub at the following URL: <https://github.com/dglgit/multicell-sims/tree/main>

## Appendix B

Selected Graphs with Individual Cellular Trajectories

 
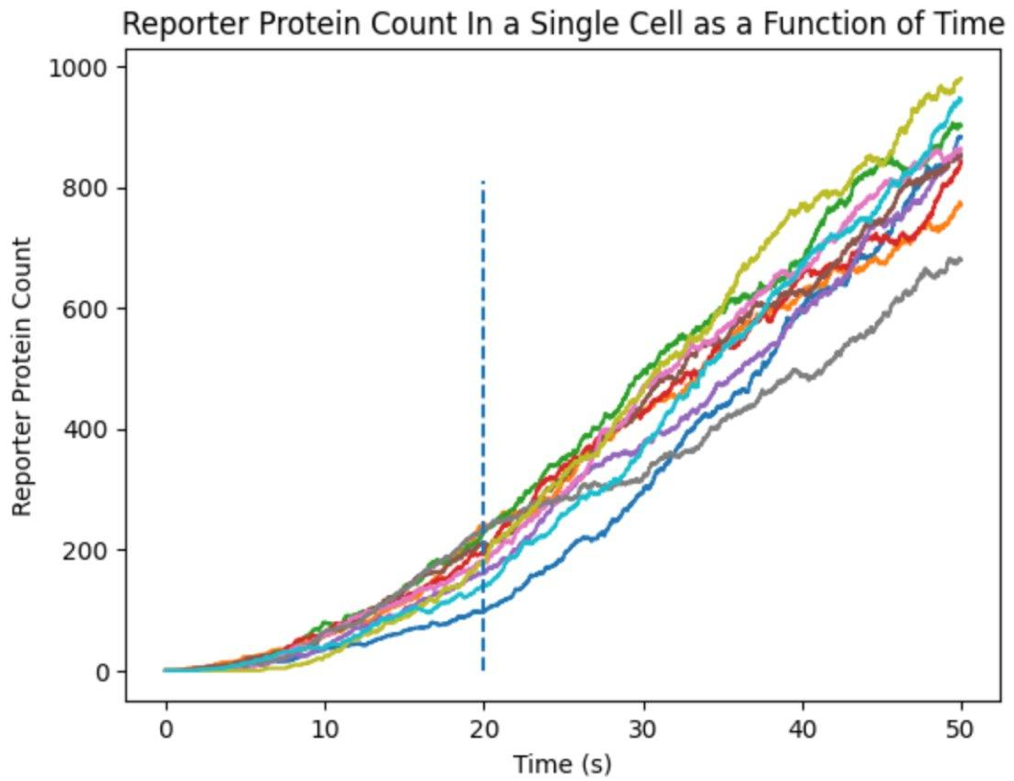


Figure B1: selected cell-level stochastic simulation with 10 cells. Each colored line represents a different cell.

  
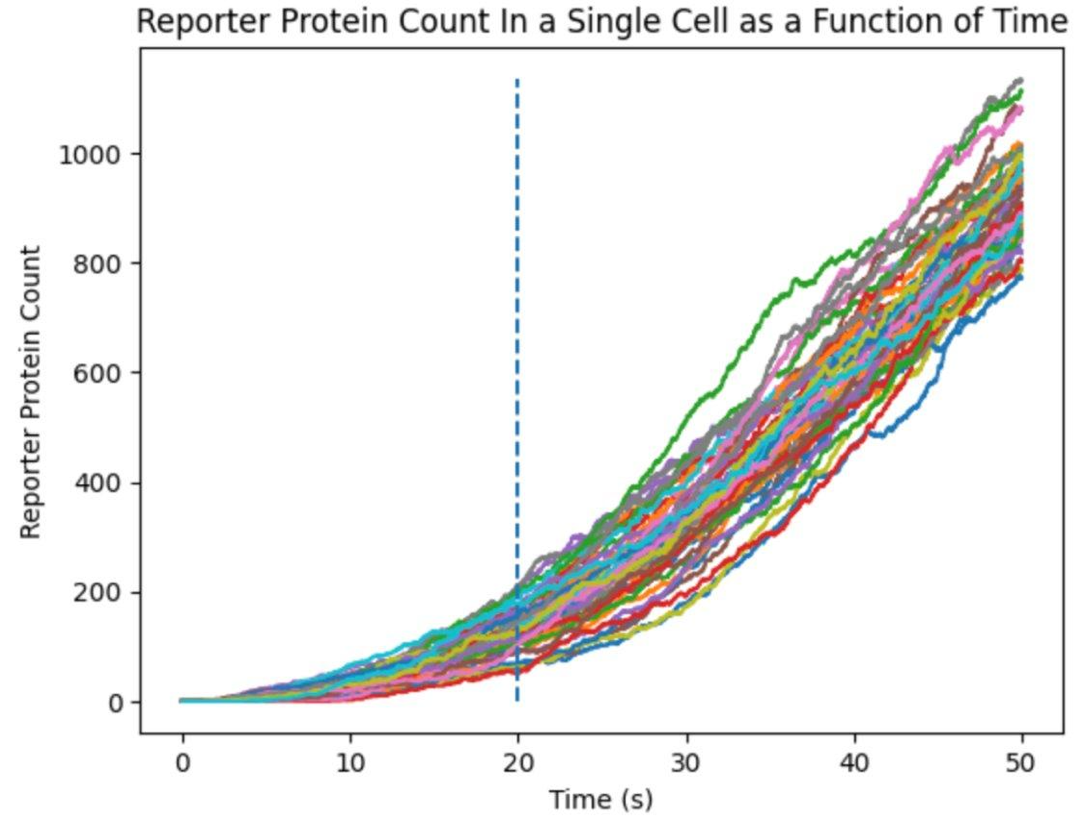


Figure B2: selected cell-level stochastic simulation with 50 cells.

 
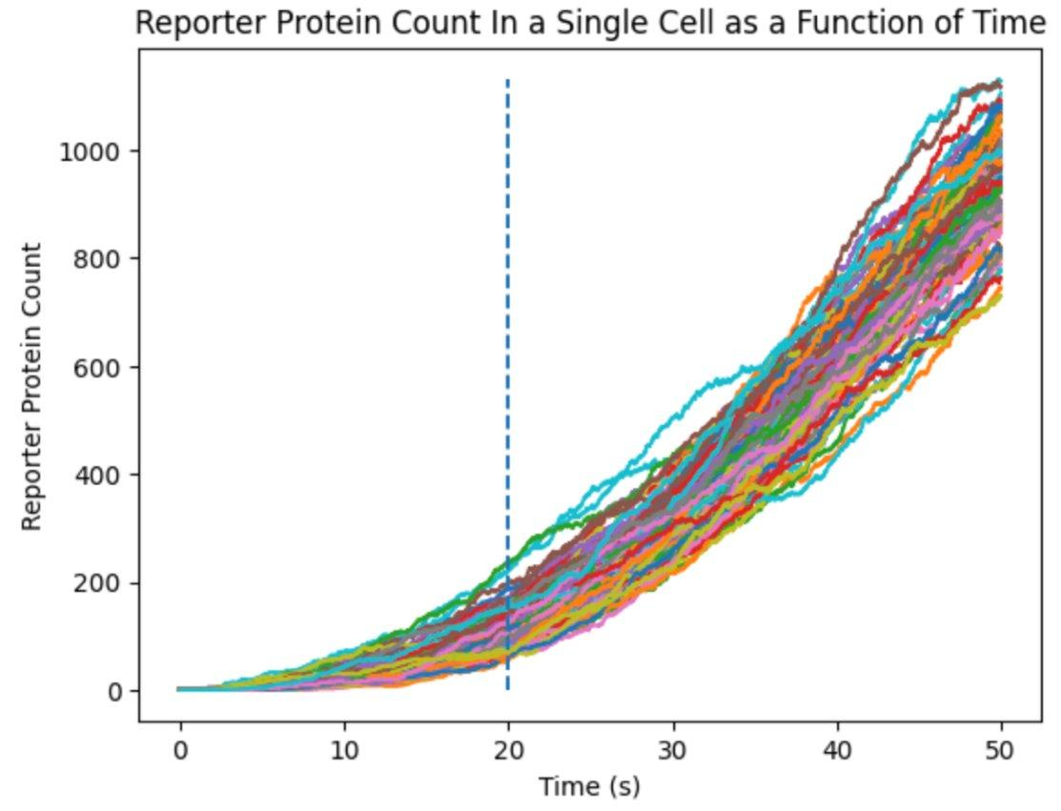


Figure B3: selected cell-level stochastic simulation with 100 cells.

## Appendix C

Comparison of Single Cellular Simulation Between Implemented Gillespie Algorithm and Virtual Cell Simulation


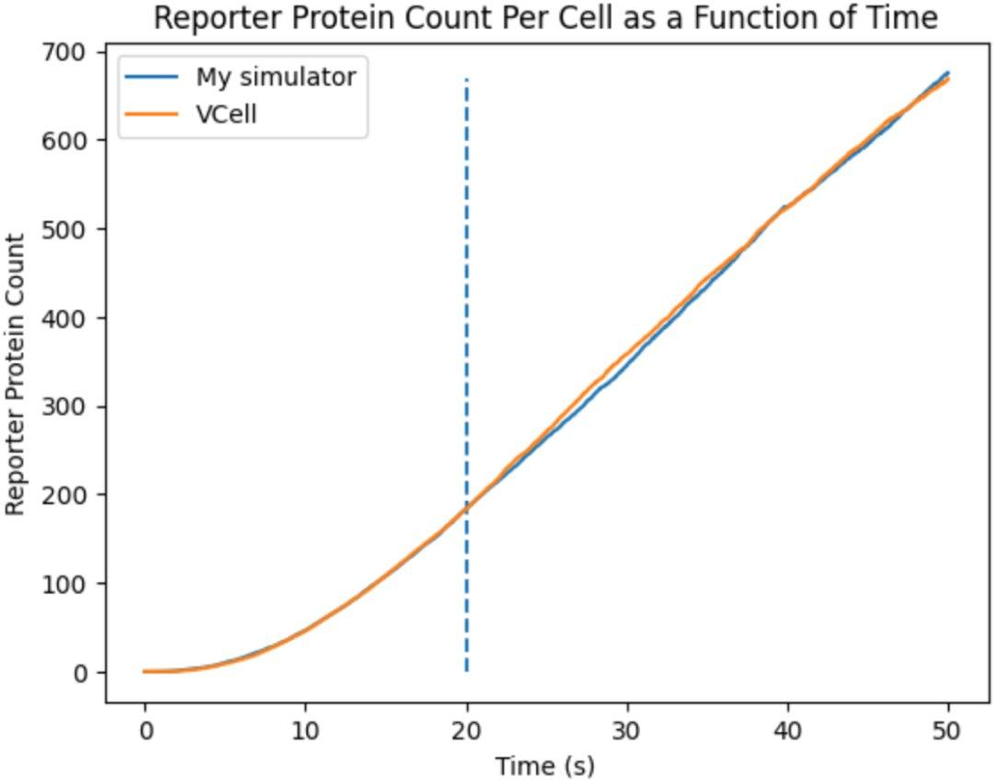


Figure C1: Graphed comparison of a reconstructed Virtual Cell simulation of the experimental construct and the simulation produced by the program written in this work. The r^2^ value for the two lines is equal to 0.997, indicating a very strong correlation.

## Appendix D

Raw Data from Each Trial

Raw trial data for average number of reporter protein molecules in each cell. The unit is the number of molecules of the reporter protein. Data link: <https://docs.google.com/spreadsheets/d/e/2PACX-1vSoK7N42hsSfVyEoZObT7gmuzuOHOLiKYX-4JbbRE5n4E1D4UbL90nDnhOvd1sb-g/pubhtml>

## Appendix E

Additional Analysis of Dilution of Extracellular Signal Across Multiple Cells


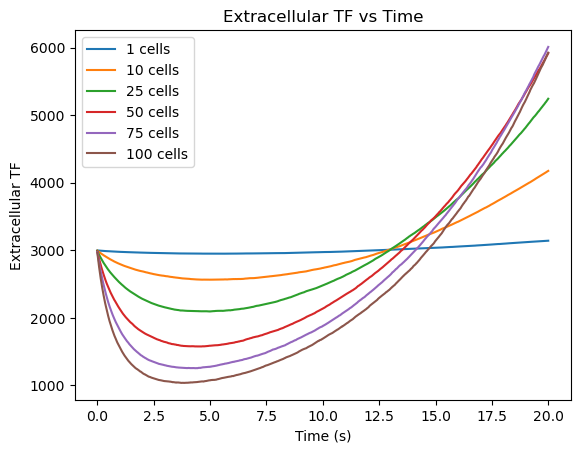


Figure E1: Plot of extracellular TF over time in a 20 second simulation.


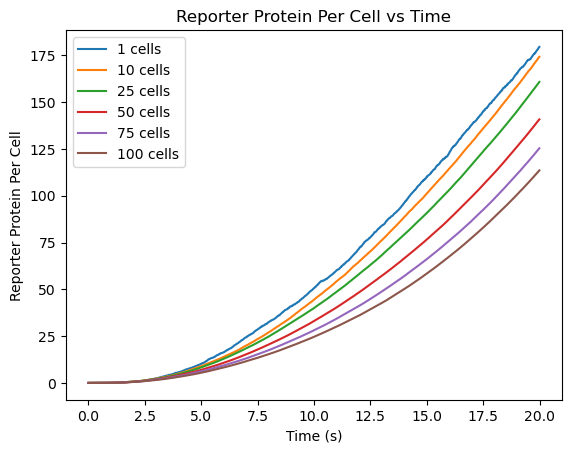


Figure 2E: plot of reporter protein per cell over time for a 20 second simulation.

As shown in Figure 1E, more cells resulted in a faster initial dip in extracellular TF, which is a proxy for how much cells are communicating with each other. This could be rationalized by the fact that the total rate of diffusion of TF out of the extracellular space and into any cell scales with the number of cells present. The initial dip translates to the staggered rates of production seen in Figure 2E.


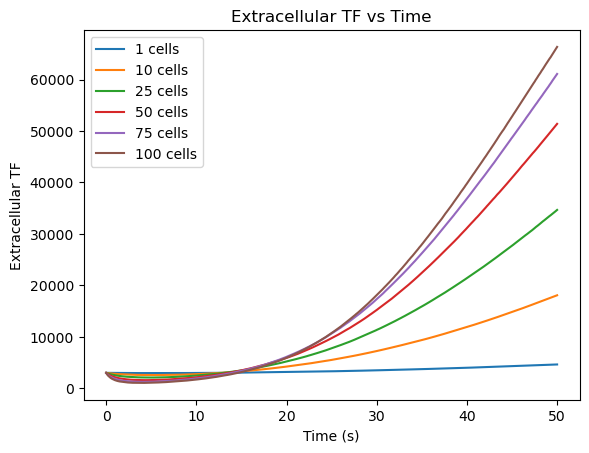


Figure E3: Plot of extracellular TF over time in a 50 second simulation.


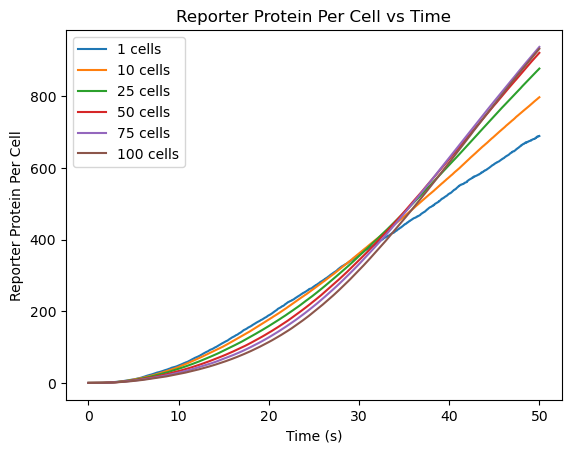


Figure E4: Reporter protein produced per cell over time in a 50 second simulation.

In Figure E3 we can see that at 50 second time scales, the initial dip flux of extracellular TF into intracellular spaces is outweighed by the increase in TF production in a positive feedback loop. The effect of this overtaking on reporter protein production can be seen in Figure 4E.
